# Magnetoencephalography abnormalities in adult mild traumatic brain injury: A systematic review

**DOI:** 10.1016/j.nicl.2021.102697

**Published:** 2021-05-08

**Authors:** Christopher M. Allen, Lloyd Halsey, Gogem Topcu, Lukas Rier, Lauren E. Gascoyne, John W Scadding, Paul L. Furlong, Benjamin T. Dunkley, Roshan das Nair, Matthew J. Brookes, Nikos Evangelou

**Affiliations:** a Mental Health and Clinical Neurosciences Academic Unit, School of Medicine, University of Nottingham, Queen's Medical Centre, Nottingham NG7 2UH, United Kingdom; b Mental Health and Clinical Neurosciences Academic Unit, School of Medicine, University of Nottingham, Jubilee Campus, Nottingham NG8 1BB, United Kingdom; c Sir Peter Mansfield Imaging Centre, School of Physics & Astronomy, University of Nottingham, University Park, Nottingham NG7 2RD, United Kingdom; d National Hospital for Neurology and Neurosurgery, Queen Square, London WC1N 3BG, United Kingdom; e College of Health and Life Sciences, Institute of Health and Neurodevelopment, Aston University, The Aston Triangle, Birmingham B4 7ET, United Kingdom; f Department of Medical Imaging, University of Toronto. 263 McCaul Street, Toronto M5T 1W7, Canada

**Keywords:** Magnetoencephalography, Mild traumatic brain injury, Systematic review

## Abstract

**Background:**

The global incidence of traumatic brain injuries is rising, with at least 80% being classified as mild. These mild injuries are not visible on routine clinical imaging. The potential clinical role of a specific imaging biomarker be it diagnostic, prognostic or directing and monitoring progress of personalised treatment and rehabilitation has driven the exploration of several new neuroimaging modalities. This systematic review examined the evidence for magnetoencephalography (MEG) to provide an imaging biomarker in mild traumatic brain injury (mTBI).

**Methods:**

Our review was prospectively registered on PROSPERO: CRD42019151387. We searched EMBASE, MEDLINE, trial registers, PsycINFO, Cochrane Library and conference abstracts and identified 37 papers describing MEG changes in mTBI eligible for inclusion. Since *meta*-analysis was not possible, based on the heterogeneity of reported outcomes, we provide a narrative synthesis of results.

**Results:**

The two most promising MEG biomarkers are excess resting state low frequency power, and widespread connectivity changes in all frequency bands. These may represent biomarkers with potential for diagnostic application, which reflect time sensitive changes, or may be capable of offering clinically relevant prognostic information. In addition, the rich data that MEG produces are well-suited to new methods of machine learning analysis, which is now being actively explored.

**Interpretation:**

MEG reveals several promising biomarkers, in the absence of structural abnormalities demonstrable with either computerised tomography or magnetic resonance imaging. This review has not identified sufficient evidence to support routine clinical use of MEG in mTBI currently. However, verifying MEG’s potential would help meet an urgent clinical need within civilian, sports and military medicine.

## Highlights

• Magnetoencephalography has higher sensitivity than clinical imaging to detect abnormalities in mild traumatic brain injury
• Excess resting state low frequency power is consistently detected following mild traumatic brain injury.
• There are widespread magnetoencephalography connectivity changes following mild traumatic brain injury.
• Machine learning techniques generate high classification accuracy when analysing magnetoencephalography data.
• Currently there is not enough evidence for routine clinical use of magnetoencephalography in mild traumatic brain injury

## 1 Introduction

Traumatic brain injury has an estimated worldwide incidence of 27 million cases annually and causes a substantial healthcare burden (James et al., 2019). At least 80% of injuries presenting to hospital are currently classified as mild traumatic brain injury (mTBI) (Excellence NIfHaC. Head injury: assessment and early management. Clinical guideline [CG176]., 2017). The global incidence of TBI is increasing, possibly due to increases in population density, population ageing, and increasing use of motor vehicles. The American Congress of Rehabilitation Medicine and later the World Health Organisation produced definitions of mTBI that are in widespread use (Head, 1993, Carroll et al., 2001). Common features include symptoms suggesting disruption of brain function following transfer of mechanical energy to the head by external forces. The severity is limited by post-traumatic amnesia of < 24 h, loss of consciousness < 30 min, and Glasgow Coma Score of 13–15 on assessment in hospital. The commonest causes of mTBI worldwide are falls and road traffic injuries (James et al., 2019). Additional causes that have attracted increasing interest in the research literature include military deployment-related blast or non-blast injuries, and sports related injuries – commonly known as concussion. The acute pathophysiology of mTBI has been shown to include axonal injury and clusters of microglial proliferation (Oppenheimer, 1968). The resultant biochemical and immunological cascade is hypothesised to leave the brain vulnerable to additional insults, pending physiological recovery (Giza and Hovda, 2014).

Post-concussion symptoms (PCS) include headache, dizziness or balance disorders, and cognitive impairments including attention, concentration, memory and speed of information processing problems. Symptoms can also include sleep disturbances, blurred vision, photosensitivity, tinnitus and neuropsychiatric symptoms including personality change, irritability, anxiety, and depression that can develop following mTBI (Bazarian et al., 1999). Whether these symptoms comprise a specific syndrome is questionable, because of their subjective nature, and that individually, some of the symptoms can occur in the healthy population or overlap with other conditions. These include anxiety, depression, and post-traumatic stress disorder (PTSD). Systematic reviews suggest group level neuropsychological cognitive testing differences disappear by three months post-mTBI (Belanger et al., 2005). This contrasts with large, prospective cohort studies, that report 50% of participants were still symptomatic on subjective measures (including cognitive complaints) at one year post-mTBI (Nelson et al., 2019, McInnes et al., 2017, Wilson et al., 2021).

Magnetoencephalography (MEG) is a functional neuroimaging technique that measures the magnetic induction produced by electrochemical current flows within the brain (Proudfoot et al., 2014). Currently sensory arrays must be cooled by liquid helium to operate, representing a significant cost; therefore, only around 200 MEG scanners were operational worldwide as of 2017 (Hari et al., 2018). However, technical innovations have allowed the development of prototype MEG sensory arrays that can operate at room temperature (Boto et al., 2018). The advantage of MEG lies in a much higher temporal resolution than functional MRI, with technical developments aimed at matching the former’s spatial resolution. The key components of the MEG signal are its amplitude and frequency. Frequency bands with clinical relevance, first defined by electroencephalography (EEG) studies are: delta 0.2 – 3 Hz, theta 4 – 7 Hz, alpha 8 – 13 Hz, beta 14 – 31 Hz, and gamma 32 – 100 Hz (Mandal et al., 2018;12:60-.). There are numerous analysis methods for interpreting MEG data, which can be recorded with the participant at rest, or performing a task. Reviewing the recorded data constitutes sensor space analysis. The overall brain signal has a peak spectral power, which at rest falls in the high alpha band over the occiput for the healthy population. Mapping the recorded signals on to an anatomical image of the brain requires inverse modelling, called source space analysis. Connectivity analysis can then be performed. This is based on the theory that spatially separate brain regions use synchronous firing of neuronal assemblies to facilitate long-range communication and the creation of a transient and dynamic task-specific network, or communication through coherence (Fries, 2005). Oscillatory amplitude envelope connectivity analysis can be used to establish the location and strength of synchronously firing neuronal populations, within and between brain regions (Brookes et al., 2016). Other network metrics seek to measure global network properties using graph theory to monitor for changes in health and disease states (van Straaten and Stam, 2013). Given the complexity of the recorded MEG data, a novel approach is to use machine-learning algorithms to classify participants, without having prior knowledge of the key discriminatory components of the MEG data (Zubarev et al., 2019). Consensus guidelines on methodology and reporting of MEG studies exist (Hari et al., 2018, Gross et al., 2013), alongside guidelines for research concerning mTBI (Carroll et al., 2001, Thompson et al., 2015).

Earlier neurophysiological research in mTBI comes from EEG studies. They demonstrated focal abnormalities in the delta and theta frequency bands as well as posterior alpha peak slowing; however, there is little evidence for correlation of either routine or quantitative EEG with clinical features of mTBI (Nuwer et al., 2005). There is an increasing incidence of civilian mTBI, growing awareness of the possible long-term consequences of sports-related concussion, and focus on optimum treatment of mTBI in the military services. Biomarkers visible on CT and standard structural MRI that can aid diagnosis or prognostication in moderate and severe injury are absent or infrequent in mTBI. Therefore, this review will focus on: 1. What changes are evident on MEG in adult mTBI? 2. Are MEG changes related to PCS in mTBI? 3. Are MEG changes related to neuropsychological test abnormalities in mTBI? 4. Are any changes related to time post-injury? 5. Do MEG changes differ according to the injury mechanism in mTBI?

## 2 Methodology

A systematic review of the literature was conducted with planned narrative synthesis, and possible meta-analysis dependent on data availability. The protocol was prospectively registered on PROSPERO CRD42019151387. A literature search of the electronic databases EMBASE, MEDLINE and PsycINFO via Ovid was conducted on 4th December 2020. The complete search strategies are listed in the supplementary material. All relevant papers published prior to the search date were included. References were screened for additional papers and searches of grey literature were conducted on Web of Science, ProQuest, World Health Organisation clinical trials registry, ISCRTN clinical trials registry and the US National Library of Medicine clinical trials registry.

After de-duplication two authors screened the 466 abstracts independently. The inclusion criteria were human research, in adults aged over 16 years, who were clinically diagnosed with mTBI according to recognised criteria with post-traumatic amnesia ≤ 24 h, Glasgow Coma Scale ≥ 13, and loss of consciousness ≤ 30 min. MEG was used as an imaging modality and comparison was made between the mTBI participants and either a normative database or a case control design was used. Outcome assessments included symptom scores, neuropsychological test scores, or clinical diagnosis. The exclusion criteria were papers not available in English, mTBI was not diagnosed by recognised criteria, paper examining pharmacological interventions, mixed diagnoses with mTBI results not published as a subgroup analysis, mixed ages with adult results not published as a subgroup analysis, and review articles, single case reports, and duplicate papers. All disagreements were resolved by discussion and 383 abstracts were rejected, leaving 83 remaining. Two authors then conducted a full text screen independently, 46 papers were rejected, leaving 37 for final inclusion in the narrative synthesis. The Scottish Intercollegiate Guidelines Network critical appraisal checklists for either case-control or cohort study designs were used to appraise risk of bias and quality of individual studies (Scotland, 2020). After review of the available data meta-analyses were not performed.

## 3 Results

### 3.1 Characteristics of included papers

**Fig. 1:**
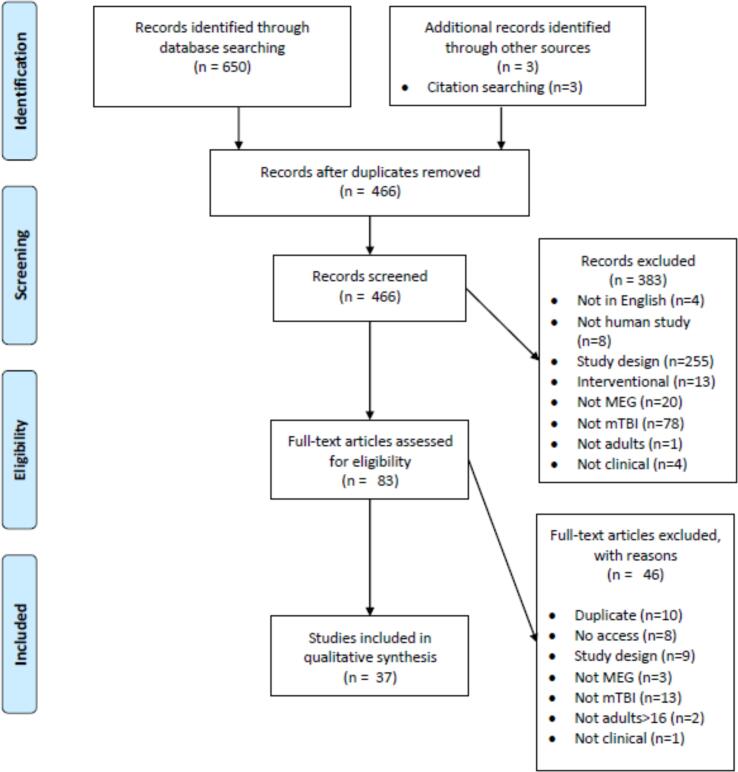
PRISMA flowchart of systematic review process.

In total, 37 papers were identified through text searching, detailed in Fig. 1. A summary of extracted study characteristics is shown in Table 1. Thirty-three papers reported a case-control design and four a cohort design. Five of the 33 case-control papers featured longitudinal MEG assessment, 13 matched participants and controls for handedness, only one reported a consecutive recruitment strategy, and none reported being prospectively registered. Orthopaedic controls were used in two of the papers, veterans or active-duty military personnel in six, healthy controls in 25, and a mix in three studies. Fifteen papers reported baseline clinical measures and 16 reported baseline years of education or estimated pre-morbid IQ.

**Table 1:** Characteristics of 37 papers included in review.

Reference	Country and study design	Study mTBI population	Mechanism of mTBI	Number of mTBI participants	Mean time post-injury (Days)	Mean age of mTBI participants (Years)	Sex of mTBI participants (% male)	Control type	Analysis type	Risk of bias
Delayed and disorganised brain activation detected with magnetoencephalography after mild traumatic brain injury (da Costa et al., 2015)	Canada, case-control	ED department, non-consecutive	Not specified	16	33	31	100	16 HC	Task-based source analysis	Highest
Low-frequency connectivity is associated with mild traumatic brain injury (Dunkley et al., 2015)	Canada, case-control	ED department, non-consecutive	7 Sports, 13 Civilian	20	32	31	100	21 HC	RS source analysis, RS connectivity analysis	Intermediate
Default mode network oscillatory coupling is increased following concussion (Dunkley et al., 2018)	Canada, case-control	ED department, non-consecutive	Not specified	26	32	31	100	24 HC	RS connectivity analysis	Lowest
Post-Traumatic stress constrains the dynamic repertoire of neural activity (Mišić et al., 2016)	Canada, case-control	ED department, non-consecutive	Not specified	20	32	31	100	20 control soldiers, 20 civilian HC, 23 soldiers with PTSD	RS source analysis, RS connectivity analysis.	Intermediate
Reduced brain connectivity and mental flexibility in mild traumatic brain injury (Pang et al., 2016)	Canada, case-control	ED department, non-consecutive	Not specified	16	33	31	100	16 HC	Task-based connectivity analysis (sensor space)	
Detecting Mild Traumatic Brain Injury Using Resting State Magnetoencephalographic Connectivity (Vakorin et al., 2016)	Canada, case-control	ED department, non-consecutive	Not specified	20	32	31	100	21 HC	RS connectivity analysis, machine learning algorithm.	Lowest
Concussion Alters the Functional Brain Processes of Visual Attention and Working Memory (Shah-Basak et al., 2018)	Canada, case-control	ED department, non-consecutive	4 Sports, 14 Civilian	18	36	30	100	19 HC	Task-based source analysis	Intermediate
Activation of dominant hemisphere association cortex during naming as a function of cognitive performance in mild traumatic brain injury: Insights into mechanisms of lexical access (Popescu et al., 2017)	USA, cohort	PCS outpatient programme	Not specified	57	1920	39	99	None	Task-based source analysis	Highest
Reduced prefrontal MEG alpha-band power in mild traumatic brain injury with associated posttraumatic stress disorder symptoms (Popescu et al., 2016)	USA, cohort	PCS outpatient programme	Not specified	32	1590	40	100	None	RS source analysis	Highest
Post-traumatic stress disorder is associated with altered modulation of prefrontal alpha band oscillations during working memory (Popescu et al., 2019)	USA, cohort	PCS outpatient programme	Not specified	35	Not specified	42	100	None	Task-based source analysis	Highest
Altered cross-frequency coupling in resting-state MEG after mild traumatic brain injury (Antonakakis et al., 2016)	USA, case-control	Texas trauma centres	2 Sports, 28 Civilian	30	Not specified	29	60	50 HC	Connectivity analysis (sensor space), machine learning algorithm	Highest
Altered rich-club and frequency-dependent subnetwork organization in mild traumatic brain injury: A MEG resting-state study (Antonakakis et al., 2017)	USA, case-control	Texas trauma centres	2 Sports, 28 Civilian	30	Not specified	29	60	50 HC	Connectivity analysis (sensor space), network metrics, machine learning algorithm	Highest
Reconfiguration of dominant coupling modes in mild traumatic brain injury mediated by delta-band activity: A resting state MEG study (Antonakakis et al., 2017)	USA, case-control	Texas trauma centres	2 Sports, 28 Civilian	30	Not specified	29	60	50 HC	Connectivity analysis (sensor space), network metrics, machine learning algorithm	Highest
Data-Driven Topological Filtering Based on Orthogonal Minimal Spanning Trees: Application to Multigroup Magnetoencephalography Resting-State Connectivity (Dimitriadis et al., 2017)	USA, case-control	Texas trauma centres	2 Sports, 28 Civilian	30	Not specified	29	60	50 HC	Network metrics, machine learning algorithms	Highest
Functional connectivity changes detected with magnetoencephalography after mild traumatic brain injury (Dimitriadis et al., 2015)	USA, case-control	Texas trauma centres	2 Sports, 29 Civilian	31	Not specified	29	58	50 HC	Connectivity analysis (sensor space), network metrics, machine learning algorithm	Highest
Improving the Detection of mTBI Via Complexity Analysis in Resting - State Magnetoencephalography (Antonakakis et al., 2016)	USA, case-control	Texas trauma centres	2 Sports, 28 Civilian	30	Not specified	29	60	50 HC	Network metrics, machine learning algorithm	Highest
Functional connectivity changes in mild traumatic brain injury assessed using magnetoencephalography (Zouridakis et al., 2012)	USA, case-control	Texas trauma centres	Not specified	10	Not specified	31	70	50 HC	Connectivity analysis (sensor space), machine learning algorithm	Highest
Magnetoencephalography slow-wave detection in patients with mild traumatic brain injury and ongoing symptoms correlated with long-term neuropsychological outcome (Robb Swan et al., 2015)	USA, case-control	TBI clinics with persistent PCS > 3 months	6 Sports, 20 Blast related, 5 Civilian	31	97	27	90	33 HC	RS source analysis	Intermediate
An automatic MEG low-frequency source imaging approach for detecting injuries in mild and moderate TBI patients with blast and non-blast causes (Huang et al., 2012)	USA, case-control	Veterans brain injury centre with persistent PCS	23 Military, 22 Civilian	45	250	28	84	44 HC	RS source analysis	Intermediate
Theta-Band Oscillations as an Indicator of Mild Traumatic Brain Injury (Kaltiainen et al., 2018)	Finland, case-control	Not specified	Not specified	26	Longitudinal	41	58	139 HC from previous study dataset	RS source analysis	Highest
Mild traumatic brain injury affects cognitive processing and modifies oscillatory brain activity during attentional tasks (Kaltiainen et al., 2019)	Finland, case-control	Not specified	4 Sports, 21 Civilian	25	Longitudinal	42	56	20 HC	Task-based sensor space and source analyses	Intermediate
Source Connectivity Analysis Can Assess Recovery of Acute Mild Traumatic Brain Injury Patients (Li et al., 2018)	USA, case-control	Not specified	Not specified	13	Longitudinal	26	54	8 orthopaedic trauma controls	RS connectivity analysis	Highest
Brain Activation Profiles in mTBI: Evidence from Combined Resting-State EEG and MEG Activity (Li et al., 2015)	USA, case-control	Not specified	Not specified	6	Not specified	28	66	5 orthopaedic trauma controls	RS analysis (sensor space)	Highest
Contrasting Effects of Posttraumatic Stress Disorder and Mild Traumatic Brain Injury on the Whole-Brain Resting-State Network: A Magnetoencephalography Study (Rowland et al., 2017)	USA, case-control	Veterans	Military	12	2265	39	100	10 HC	Network metrics	Highest
Increased Small-World Network Topology Following Deployment-Acquired Traumatic Brain Injury Associated with the Development of Post-Traumatic Stress Disorder (Rowland et al., 2018)	USA, cohort	Veterans	Military	16	4138	40	100	None	Network metrics	Highest
MEG Working Memory N-Back Task Reveals Functional Deficits in Combat-Related Mild Traumatic Brain Injury (Huang et al., 2019)	USA, case-control	Veterans or active-duty military personnel with persistent PCS	Military	25	315	27	100	20 veterans or active-duty military personnel	Task-based source analysis	Lowest
Marked Increases in Resting-State MEG Gamma-Band Activity in Combat-Related Mild Traumatic Brain Injury (Huang et al., 2019)	USA, case-control	Veterans or active-duty military personnel with persistent PCS	Military	25	594	28	100	35 veterans or active-duty military personnel	RS source analysis	Highest
Single-subject-based whole-brain MEG slow-wave imaging approach for detecting abnormality in patients with mild traumatic brain injury (Huang et al., 2014)	USA, case-control	Persistent PCS	36 Military, 48 Civilian	84	265	29	83	11 veterans or active-duty military personnel 68 civilian HC	RS source analysis	Highest
Resting-State Magnetoencephalography Reveals Different Patterns of Aberrant Functional Connectivity in Combat-Related Mild Traumatic Brain Injury (Huang et al., 2017)	USA, case-control	Veterans or active-duty military personnel	26 Military	26	508	28	100	22 veterans or active-duty military personnel	RS connectivity analysis	Highest
Integrated imaging approach with MEG and DTI to detect mild traumatic brain injury in military and civilian patients (Huang et al., 2009)	USA, case-control	Persistent PCS	4 Sports, 4 Military, 2 Civilian	10	353	25	90	14 HC	RS source analysis.	Highest
Attentional dysfunction and recovery in concussion: effects on the P300m and contingent magnetic variation (Petley et al., 2018)	Canada, case-control	Consecutive ED mTBI patients	2 Sports, 11 Civilian	13	Longitudinal	26	31	13 HC	Task-based ERFs	Highest
Complexity analysis of resting state magnetoencephalography activity in traumatic brain injury patients (Luo et al., 2013)	USA, case-control	Not specified	15 Military, 3 Civilian	18	1859	29	100	18 HC	Network metrics	Highest
Filling in the gaps: Anticipatory control of eye movements in chronic mild traumatic brain injury (Diwakar et al., 2015)	USA, case-control	mTBI clinic or neurology referrals with persistent PCS	13 Sports, 12 Civilian	25	968	33	84	25 HC including from other studies	Task-based source analysis	Highest
Objective documentation of traumatic brain injury subsequent to mild head trauma: Multimodal brain imaging with MEG, SPECT, and MRI (Lewine et al., 2007)	USA, cohort	Outpatient clinics with persistent PCS > 1 year	30 Civilian	30	1011	38	53	None	RS source analysis	Highest
Neuromagnetic assessment of pathophysiologic brain activity induced by minor head trauma (Lewine et al., 1999)	USA, case-control, longitudinal	mTBI with or without PCS	Not specified	30	345	36	60	20 HC	RS source analysis	Highest
Aberrant Whole-Brain Transitions and Dynamics of Spontaneous Network Microstates in Mild Traumatic Brain Injury (Antonakakis et al., 2020)	USA, case-control	Texas trauma centres	2 Sports, 28 various	30	Not specified	29	60	50 HC	Network metrics	Highest
Local and large-scale beta oscillatory dysfunction in males with mild traumatic brain injury (Zhang et al., 2020)	Canada, case-control	Non-consecutive ED mTBI patients	12 Sports, 15 Civilian	27	39	30	100	23 HC	RS source analysis, RS connectivity analysis	Intermediate

Twenty-five papers examined a civilian population with mixed mechanisms of injury, in five papers the population recruited from was unclear. Five papers examined a military population with two of these specifically focussed on blast injury. Two papers include both military personnel and civilians. Ten of the papers recruited only patients with mTBI and persisting PCS. The study sizes ranged from six to 84 participants with mTBI. Mean time between injury and MEG assessment ranged from six days to 13 years but was unreported in nine papers. There was a male bias in the mTBI population of all included papers, with 17 reporting exclusively male participants. The mean mTBI sample age ranged from 25 to 42 years. Year of publication spanned 1999 to 2020.

Ten papers reported sensor space analyses while the remaining 27 reported findings after source reconstruction. Fourteen papers presented analysis of resting state spectral power. Seventeen papers presented connectivity analyses or report network metrics. Nine papers presented analyses of task-based MEG recordings. Symptom severity was correlated with MEG findings in twelve papers, and neuropsychological test scores in five papers. Thirteen papers attempted classification metrics, most of these being machine-learning algorithms. Several papers reported multi-modal imaging, but only two presented associations between MRI abnormalities and their MEG findings.

### 3.2 Spectral power analysis

MEG demonstrated improved ability to detect spectral power differences over EEG when utilising multimodal imaging (Li et al., 2015). The most common finding was increased power in the delta frequency band of the MEG signal in mTBI participants relative to controls, reported in eight of the 14 papers that described spectral power analysis, as shown in Table 2 (Li et al., 2015, Dunkley et al., 2015, Robb Swan et al., 2015, Huang et al., 2012, Huang et al., 2014, Huang et al., 2009, Lewine et al., 2007, Lewine et al., 1999). The location of this abnormal delta frequency band activity was variable. The most likely sites were within the temporal, frontal, and parietal lobes. Huang et al. used a voxel-based analysis to show that any individual cortical voxel had a low (5–15%) likelihood of abnormal delta generation, but the commonest areas affected in their study were bilateral dorsolateral and ventral pre-frontal cortices, frontal poles, inferior temporal lobes, and the cerebellum (Huang et al., 2014). The occipital lobes were noted to be least likely to have excess delta power in mTBI participants compared to controls in three papers (Li et al., 2015, Huang et al., 2014, Lewine et al., 2007). Antonakakis et al. was the only paper to report that controls had increased power in the delta frequency band over the frontal region compared to mTBI participants (Antonakakis et al., 2016). They calculated relative power in sensor space, and instead showed that theta and alpha frequency bands had higher power in mTBI participants compared to controls over the frontal region. Four papers reported mTBI participants had an increased power in the theta frequency band relative to controls (Li et al., 2015, Dunkley et al., 2015, Antonakakis et al., 2016, Kaltiainen et al., 2018), and the most likely sites were the temporal lobes and subcortical areas. Some studies combined delta and theta to assess for excess low frequency activity (LFA) in mTBI (Kaltiainen et al., 2018). This review did not assess the specificity of these changes; there is evidence that other conditions, e.g. Alzheimer’s disease demonstrate excess LFA on EEG (Hamm et al., 2015).

**Table 2:** Summary of spectral power analysis, with number of mTBI participants (n), and risk of bias assessment.

Frequency band	Reduced in mTBIrelative to controls	Neutral	Increased in mTBIrelative to controls
Delta	(Antonakakis, Dimitriadis et al. 2016) (30 – highest)	(Zhang, Safar et al. 2020)(27 – intermediate)	(Lewine, Davis et al. 1999)(30 – highest)(Lewine, Davis et al. 2007)(30 – highest)(Huang, Theilmann et al. 2009)(10 – highest)(Huang, Nichols et al. 2012)(45 – intermediate)(Huang, Nichols et al. 2014)(84 – highest)(Dunkley, Da Costa et al., 2015)(45 – intermediate)(Li, Pagnotta et al. 2015)(31 – intermediate)(Swan, Nichols et al. 2015)(31 – intermediate)
Theta		(Zhang, Safar et al. 2020)(27 – intermediate)	(Antonakakis, Dimitriadis et al. 2016)(30 – highest)(Dunkley, Da Costa et al., 2015)(45 – intermediate)(Kaltiainen, Helle et al. 2018)(26 – highest)(Li, Pagnotta et al. 2015)(31 – intermediate)
Alpha	(Dunkley, Da Costa et al., 2015)(45 – intermediate)(Popescu, Hughes et al. 2016)(32 – highest)		(Antonakakis, Dimitriadis et al. 2016)(30 – highest)(Li, Pagnotta et al. 2015)(31 – intermediate)(Misic, Dunkley et al. 2016)(20 – intermediate)
Beta	(Zhang, Safar et al. 2020)(27 – intermediate)		
Gamma	(Misic, Dunkley et al. 2016)(20 – intermediate)		(Huang, Huang et al. 2019)(25 – highest)

The alpha frequency band was reported to show increased power in mTBI participants compared to controls in three papers (Li et al., 2015, Antonakakis et al., 2016, Mišić et al., 2016)and the opposite relationship in two papers (Dunkley et al., 2015, Popescu et al., 2016). The latter two papers suggest that an increased power in LFA and a decrease in alpha frequency band power represents a slowing of alpha activity in mTBI. However, Mišić et al. noted an increased power in the alpha frequency band and decreased power in the gamma frequency band in civilian mTBI versus both civilian controls and military personnel, some of whom had PTSD (Mišić et al., 2016). Only one paper reported significant differences in the beta frequency band. Dunkley et al. found beta power to be significantly reduced in mTBI compared to controls in the frontal and temporal lobes (Zhang et al., 2020). Huang et al. reported that in military mTBI participants with chronic PCS there was widespread increased power in the gamma frequency band relative to military controls (Huang et al., 1991).

Kaltiainen et al. noted that only MRI T2 hyperintense lesions within 3 cm of the cortex were associated with aberrant theta frequency band activity (Kaltiainen et al., 2018). Similarly, Huang et al. showed in 10 mTBI patients with persistent post-concussive symptoms that aberrant gamma frequency band activity was associated with nearby non-major white matter tract damage, identified by decreased fractional anisotropy with DTI (Huang et al., 2009).

### 3.3 Connectivity analysis

Combining both intra and cross-frequency analyses the most frequently reported band specific connectivity analysis was in the delta frequency band, in nine of the 17 papers. Of these, three reported an increase in delta frequency band connectivity in participants with mTBI relative to controls (Dunkley et al., 2015, Li et al., 2018, Huang et al., 2017bb), and two reported a decrease (Antonakakis et al., 2016, Vakorin et al., 2016). Four papers reported their findings using an alternative network metric, such as complexity and these will be discussed at the end of this section (Antonakakis et al., 2017, Antonakakis et al., 2017, Antonakakis et al., 2016, Dimitriadis et al., 2017). The three papers reporting a relative increase in mTBI participants each noted this change in different regions of the frontal and temporal lobes. The putamen was noted to be implicated in two of the papers. None reported an increased connectivity in the occipital lobes. Of the two papers reporting decreased delta frequency band connectivity, one reported this over bilateral frontal areas in sensor space (Antonakakis et al., 2016). The other reported decreased connectivity to and from the occipital lobe in mTBI participants relative to controls (Vakorin et al., 2016). Four papers reported an increase in the theta frequency band connectivity (Dunkley et al., 2015, Li et al., 2018, Rowland et al., 2017, Luo et al., 2013), while none reported a decrease. This includes three papers that also reported an increase in the delta frequency band connectivity, with similar brain locations found to be responsible for both.

Alpha frequency band connectivity analysis was reported in seven papers. Four papers from the same group reported an increase in mTBI participants relative to controls (Dunkley et al., 2015, Dunkley et al., 2018, Mišić et al., 2016, Vakorin et al., 2016). One paper showed a non-significant decrease (Li et al., 2018), and two used alternative network metrics (Antonakakis et al., 2017, Dimitriadis et al., 2015). The most frequent locations to detect an increased connectivity were the frontal and then temporal lobes. Dunkley et al. examined both the default mode and motor networks in the resting state and found an increased connectivity in these networks in mTBI participants (Dunkley et al., 2018).

Beta frequency band connectivity was reported in five papers. Three reported an increase (Antonakakis et al., 2016, Huang et al., 2017bb, Dunkley et al., 2018), in the frontal and temporal lobes of mTBI participants relative to controls and one paper noted this was due to significant cross frequency coupling between the beta and high gamma frequency bands (Antonakakis et al., 2016). One paper reported a reduction in beta frequency band connectivity in mTBI participants relative to controls, with the most marked reduction in the bilateral somatosensory and motor cortices (Zhang et al., 2020). One paper reported alternative network metrics (Antonakakis et al., 2017). Gamma frequency band connectivity was reported in six papers, with three reporting an increased connectivity, mostly in the frontal lobes in mTBI participants relative to controls (Antonakakis et al., 2016, Huang et al., 2017bb, Dunkley et al., 2018). Two papers reported the opposite, with one finding that it was an increased high gamma functional network that most accurately distinguished mTBI participants from both controls and participants with PTSD (Mišić et al., 2016, Vakorin et al., 2016). One paper reported alternative network metrics in isolation (Antonakakis et al., 2017).

Alternative network metrics included calculating coefficients of: small-worldness (Antonakakis et al., 2017, Rowland et al., 2017), rich club nodes (Antonakakis et al., 2017, Antonakakis et al., 2017, Dimitriadis et al., 2017), efficiency (Antonakakis et al., 2016, Dimitriadis et al., 2017, Dimitriadis et al., 2015, Zouridakis et al., 2012), and complexity (Antonakakis et al., 2016, Luo et al., 2013). Summarising these results is challenging, given the variability of analysis methods, and given few findings were replicated. Many used a data driven machine-learning approach to define differences between participants with mTBI and controls and quoted high precision within their own training datasets. Three papers from the same research group described a hypersynchronised delta frequency band modulated rich club network and lower global efficiency in mTBI participants relative to controls (Antonakakis et al., 2017, Antonakakis et al., 2017, Dimitriadis et al., 2017).

### 3.4 Task-based analysis

Of the nine papers that included task-based analyses; three assessed working memory (Shah-Basak et al., 2018, Popescu et al., 2019, Huang et al., 2019cc), two set-shifting (da Costa et al., 2015, Pang et al., 2016), and one visual attention (Petley et al., 2018), visual tracking (Diwakar et al., 2015), picture naming (Popescu et al., 2017), and auditory information processing (Kaltiainen et al., 2019). These tasks were performed during the MEG recording, while the analyses above only used resting state data. Only one paper performed a connectivity analysis (Pang et al., 2016), while the rest performed spectral power analyses. The working memory tasks showed left lingual gyrus hyperactivation, as well as asymmetry of hippocampal activation (Shah-Basak et al., 2018), and bilateral frontal pole hyperactivation, in all frequency bands in mTBI participants relative to controls (Huang et al., 2019). However, Popescu et al. found a relative reduction in alpha frequency band power in the left rostral middle frontal region was correlated with task performance (Popescu et al., 2019). This was more strongly associated with PTSD symptom severity evaluated using the Post Traumatic Stress Disorder Checklist-Military (PCL-M), than the severity of mTBI in their cohort study.

In the set-shifting tasks, mTBI participants had longer reaction times and poorer performance in the extradimensional shift condition compared to controls. However, both set-shifting conditions showed mTBI participants had an aberrant sequence of brain area activation. This was significant in the right frontal and bilateral parietal lobes (da Costa et al., 2015). The same group showed that connectivity between the occipital lobes and the rest of the brain in the alpha frequency band was reduced in mTBI participants compared to controls (Pang et al., 2016). Petley et al. showed reduced global field strength and delayed reaction times in a small sample of mTBI participants compared to controls during a visual attention task (Petley et al., 2018). Visual tracking of an intermittently obscured target showed lower performance in mTBI participants and was associated with widespread relative changes in beta frequency band power compared to controls (Diwakar et al., 2015). During picture naming there was a reduction in the amplitude of the event-related MEG signal in the dominant hemisphere association areas in those of the cohort whose memory test results were poorest (Popescu et al., 2017). Kaltiainen et al. found altered activation globally in the alpha frequency band during a paced auditory serial addition test in mTBI participants compared to controls (Kaltiainen et al., 2019).

### 3.5 Clinical outcome and MEG results

Five papers reported the correlation between their MEG results and clinical interview results or symptom questionnaire scores, as a surrogate for clinical outcome. Two papers reported the sum of all regions with excess LFA positively correlated with symptom score on the Head Injury Symptom Checklist and symptom severity in a structured clinical interview, respectively (Huang et al., 2012, Lewine et al., 1999). On the contrary, two papers commented specifically that they did not find a significant correlation between MEG abnormalities and mTBI symptoms. This included resting state LFA not correlating with symptoms as recorded by the European Brain Injury Questionnaire (Lewine et al., 2007), and theta frequency band activity not correlating with symptom score on the Rivermead Post-Concussion Symptom Questionnaire (Kaltiainen et al., 2018). Dunkley et al. reported increased connectivity in the alpha and gamma frequency bands within the default mode network positively correlated with symptom score on the Sports Concussion Assessment Tool 2 (Dunkley et al., 2018).

There can be diagnostic uncertainty when attempting to differentiate PCS and PTSD. While not the focus of this review, four of the included papers reported correlations between their MEG results, predominantly in the alpha frequency band and co-morbid PTSD symptoms (Huang et al., 2014, Popescu et al., 2016, Popescu et al., 2019, Rowland et al., 2018). Popescu et al. reported lower power frontally in the resting state alpha frequency band, in those who screened positive for PTSD with the PCL-M, compared to those who did not, as well as those who had loss of consciousness associated with their mTBI (Popescu et al., 2016). During a working memory task frontal alpha frequency band power negatively correlated with symptom score (Popescu et al., 2019). Rowland et al. did not find a correlation with symptom scores; however, they did show a shift in connectivity from the alpha to theta frequency bands in both mTBI and PTSD (Rowland et al., 2017). There were few network-level differences between the mTBI, PTSD and dual diagnosis groups in this study in the alpha frequency band, however when considering all frequency bands, the mTBI group had increased small-worldness and the PTSD group had reduced small-worldness. The same group replicated their findings of increased small-worldness when participants had PTSD detected using the Clinician-Administered PTSD Scale 5 in addition to mTBI (Rowland et al., 2018).

Three of the included papers reported on the correlation between MEG findings and symptoms of depression or anxiety (Dunkley et al., 2015, Huang et al., 2014, Popescu et al., 2017). Huang et al. reported that delta frequency power in the anterior cingulate cortex correlated with depressive symptoms recorded using a modified Head Injury Symptoms Checklist (Huang et al., 2014). Dunkley et al. reported alpha frequency connectivity between left occipital and bilateral temporal and subcortical regions was positively correlated with Patient Health Questionnaire 9 and Generalised Anxiety Disorder 7 score (Dunkley et al., 2015). Yet, Popescu et al. reported no correlation between global spectral power and either of these scores (Popescu et al., 2017). Major depressive disorder, independent of mTBI, has been associated with a global excess of LFA in EEG studies (Alamian et al., 2017, Newson and Thiagarajan, 2019). Huang et al. reported trouble concentrating was associated with increased delta frequency power in the right orbitofrontal cortex and Dunkley et al. reported a positive correlation between low frequency connectivity and inattention scores on Conner’s Comprehensive Behaviour Rating Scale (Dunkley et al., 2015, Huang et al., 2014).

### 3.6 Neuropsychological testing and MEG results

There was marked variability in approach when correlating MEG data with neuropsychological testing data. Some papers used resting state data, while others used task specific data, e.g., from an N-back working memory task and both spectral power and connectivity analyses were used. The most reported neuropsychological assessments were the Trail Making Test Part B within the Delis Kaplan Executive Function Score (DKEFS), and the Digit Symbol Coding task within the Weschler Adult Intelligence Scale. Four papers reported correlations between these test scores and either power or connectivity of specific frequency bands in the frontal MEG results (Robb Swan et al., 2015, Huang et al., 2019aa, Huang et al., 2017bb, Huang et al., 2019cc). For the Trail Making Test Part B the right dorsolateral prefrontal cortex power in all frequency bands (Huang et al., 2019), and left ventrolateral prefrontal cortex beta frequency band functional connectivity (Huang et al., 2017), were negatively correlated with test performance. LFA power in the frontal poles and right precentral gyrus were also reported to be negatively correlated with test performance (Robb Swan et al., 2015). Finally, power in the gamma frequency band in the right supplementary motor area was negatively correlated with test performance and distinguished between mTBI participants and controls (Huang et al., 2019).

For the Digit Symbol Coding task, the right prefrontal cortex power in all frequency bands and low frequency power in right temporal gyri were negatively correlated with test performance (Robb Swan et al., 2015, Huang et al., 2019cc). Huang et al. found widespread negative correlations between gamma frequency band power and test performance (Huang et al., 2019). While the left superior parietal lobe, right precentral gyrus and left frontal pole LFA were positively correlated with test performance (Robb Swan et al., 2015). Left ventrolateral prefrontal cortex beta band connectivity was also positively correlated with test performance (Huang et al., 2017). Spectral power in the frontal poles, left superior parietal lobe gamma frequency band power and functional connectivity of the beta frequency band in the left ventrolateral prefrontal cortex were negatively correlated with performance of the letter fluency subtest within the DKEFS by the same author (Huang et al., 2019aa, Huang et al., 2017bb, Huang et al., 2019cc).

### 3.7 Diagnostic application

Fifteen papers described methods to determine participant classification between mTBI participants and controls. From the reports, it is unclear if any of these used methods that were set prospectively, prior to data collection. Four of these used resting state LFA (Lewine et al., 1999, Kaltiainen et al., 2018, Huang et al., 2012, Huang et al., 2014). Lewine et al. demonstrated the potential role of MEG in 1999 when they reported a sensitivity of 65% for excess LFA in mTBI participants with persistent PCS. This test had a false positive rate of 5% in healthy controls, and 10% of mTBI participants without persistent PCS tested positive (Lewine et al., 1999). Kaltiainen et al more recently showed a sensitivity of 30% in a symptomatic subacute mTBI sample, with a false positive rate in healthy controls of 1% (Kaltiainen et al., 2018). Huang et al. reported a significant increase in sensitivity by considering normalised power on an individual voxel, not whole brain basis. They reported sensitivities of 85% and 87% in symptomatic mTBI participants with specificities of 100% (Huang et al., 2012, Huang et al., 2014). The cut-off threshold was set after data processing to achieve this maximum specificity for both papers.

Ten papers applied a machine learning approach to distinguish the connectivity analysis of mTBI participants from controls (Antonakakis et al., 2016, Antonakakis et al., 2017, Antonakakis et al., 2017, Antonakakis et al., 2016, Zhang et al., 2020, Dimitriadis et al., 2015, Dimitriadis et al., 2017, Zouridakis et al., 2012, Vakorin et al., 2016). Most papers did not split their data into model training and test sets, and subsequently reported extremely high, possibly over-fitted performance. Diwakar et al. used a novel approach, combining MEG features with task performance and neuropsychological testing results to achieve a 94% classification accuracy in a chronic symptomatic mTBI cohort compared to healthy controls (Diwakar et al., 2015).

### 3.8 Time post-injury and MEG results

While the mean time between MEG assessment and injury ranged from six days to 11 years in the papers incorporated in this systematic review, five papers included repeat MEG imaging sessions (Lewine et al., 1999, Kaltiainen et al., 2018, Kaltiainen et al., 2019, Li et al., 2018, Petley et al., 2018). Three papers showed the incidence of abnormal LFA dropped as the interval between MEG imaging and injury increased, suggesting this represents an acute to subacute marker of injury that may also be linked to recovery (Lewine et al., 1999, Kaltiainen et al., 2018, Li et al., 2018). However, when considering all papers that reported LFA as able to differentiate mTBI participants from controls, the mean time to scanning ranges from one week to 33 months. Given this discrepancy between longitudinal and cross-sectional study designs, it is not possible to ascertain whether excess LFA resolution is associated with symptomatic recovery from mTBI. Two of the papers with serial MEG imaging found that differences in task-based alpha frequency band power and event related potentials differentiated mTBI participants and controls acutely and 3–6 months later, suggesting the MEG abnormalities persist (Petley et al., 2018, Kaltiainen et al., 2019). Both papers noted that only small subsets of their samples returned for serial MEG sessions, which may have biased their results.

### 3.9 Mechanism of injury and MEG results

Individual studies did not report the ability to detect differing MEG abnormalities dependant on the mechanism of injury. The mechanisms were divided into sports-related concussion, any other civilian injury, or those suffered by military personnel, which could be further split into blast (from explosive blast waves) and non-blast trauma. In total 16 papers reported mTBI participants from more than one of these groups, though small sample sizes may have led to underpowered comparison.

### 3.10 Risk of bias

Three papers were sufficiently detailed to complete at least two thirds of the relevant Scottish Intercollegiate Guidelines Network critical appraisal checklist and judged to be at the lowest risk of bias (Vakorin et al., 2016, Dunkley et al., 2018, Huang et al., 2019cc). Seven papers were judged at intermediate risk of bias (Mišić et al., 2016, Zhang et al., 2020, Shah-Basak et al., 2018, Kaltiainen et al., 2019, Dunkley et al., 2015, Robb Swan et al., 2015, Huang et al., 2012), and the remaining 18 at high risk of bias. Frequent concerns for potential bias in the 32 case control studies were lack of clinical description of participants and adequate screening of controls to avoid inclusion of cases with many papers not reporting exclusion criteria. In addition, there was often inadequate controlling for potential confounders, and lack of a clearly defined prospective research question. Within the five cohort studies, areas of potential bias included a lack of clearly defined pre-specified outcomes and not reporting on blinding when performing the analysis. Another potential concern is the possibility of overlapping clinical samples, or unacknowledged re-analysis of previous datasets, which may lessen the impact of the entire field.

## 4 Discussion

This review has identified that while MEG has demonstrated clear promise as a functional neuroimaging modality, it is not yet a diagnostic or prognostic clinical tool in mTBI of sufficient sensitivity and specificity. However, MEG is one of the most sensitive imaging modalities for the evaluation of mTBI, considering the very low sensitivity of CT, structural MRI and EEG. There is growing consensus around key features such as an increase in LFA power and widespread connectivity changes following mTBI. The consistently high prevalence of MEG abnormalities across several studies, and the initial successes of AI algorithms to classify participants, implies that MEG is one of the most sensitive neuroimaging modalities to investigate this condition. Future work should concentrate on harmonising biomarkers and data analysis methods, so that different groups can generate a robust evidence base quickly. Harmonisation should also aim to build on the current published longitudinal studies to establish the natural history of these changes in the weeks, months and years following injury. Current evidence indicates that task-based MEG data, with cognitive loading, are also an important tool to improve our understanding of the impact of mTBI on neural activity and could possibly play a role in guiding therapeutic interventions.

Increases in LFA power have been reported with a frontal predilection. This correlates with acute changes in mTBI in the corpus callosum seen using DTI. In addition, when abnormal LFA and MRI lesions appear to co-localise, it suggests that LFA may arise from partial cortical deafferentation (Gloor et al., 1977). Despite this, LFA is known to be non-specific, occurring in other conditions such as depression or secondary to medication use (Newson and Thiagarajan, 2019). Differences in measurement techniques may explain the variation in reported prevalence of the abnormal LFA, so despite two studies suggesting it can resolve with time, and some evidence of association with symptoms or neuropsychology test results, its role as a diagnostic or prognostic marker is yet to be determined. The heterogeneity of available neuropsychological tests and symptom scoring tools additionally limits the robustness of this conclusion. The findings reported in this systematic review are often the result of group level comparisons, but two papers of intermediate risk of bias differentiated between their chronic PCS participants and controls on a single participant level with high accuracy. However, none of the included studies met the criteria of a high quality prospective clinical diagnostic test accuracy study.

Many papers have examined the role of network metrics, connectivity, and machine learning. There is a lack of methodological homogeneity across papers, and studies have not addressed the direction of observed effects. However, an increase in delta and theta connectivity is reported, including in four of the papers at minimum or intermediate risk of bias. Authors have suggested that these effects are reflective of plasticity in recovery, and symptoms may be related to an inability to deactivate the default mode network. Network metric studies often used machine learning to report high levels of classification accuracy, but frequently used convenience samples of unmatched controls, making them vulnerable to spectrum bias. While not yet being clinically useful, this shows a potential role for machine learning, which should be explored further.

The most common risks of bias identified in this review related to clarity of outcome measures, likely retrospective unblinded analysis and a lack of clinical description of participants, leading to the possibility of confounding. Most studies were small, the largest included 84 participants with mTBI. Additionally, the analysis performed was heterogeneous, with the most common type of analysis (a connectivity analysis) being performed in only 17 of the 37 included papers. There was a wide intra-study and inter-study range of time interval between injury and MEG scanning, which may mask some of the temporal evolution of MEG changes following mTBI.

For future studies, collaboration across sites should be encouraged. This will increase sample size and power, and prospective registration with clear quantifiable outcome measures would limit bias. These should align with recommended core outcome sets for mTBI research (Carroll et al., 2001, Thompson et al., 2015). An appropriately matched trauma-exposed control group should be used. This is especially important if the intention is to apply machine learning techniques. This would be more representative of the population that mTBI participants are drawn from, ensure machine learning only detects features related to mTBI, and will reveal pragmatic false positive rates, which would be more applicable to clinical settings. To further limit bias, the baseline characteristics of both control and case samples should be clearly stated and ideally matched, given that this is known to influence MEG findings. Exclusion criteria should be well defined, dropout rates stated, and impact on results considered. Regarding the application of machine learning within these studies, training and test populations should be separate to avoid over-fitting. More importance should be given to repeatability, ideally across different scanners and clinical settings.

## 5 Limitations

The major limitations of this review were being unable to resolve its broad questions into quantitative measures and the inability to perform a *meta*-analysis of MEG data, based on the available literature. For example, different mechanisms of injury could not be differentiated by MEG within individual studies. If this data could be pooled, and assessed with a pre-specified analysis method, we could definitively answer this question. This issue arises because of the broad definition of mTBI, the complex nature of the MEG datasets and variety of analysis methods available and reported. It is likely that a pooling of original study datasets will be required to overcome this, but this was beyond the scope of this review. The review’s strengths include the prospectively registered systematic design and independent rating of papers, which should limit the risk of bias in its conclusions. Additionally, this review has identified and made recommendations to improve study methodology, frequently judged suboptimal by clinical critical appraisal tools.

## 6 Conclusion

To the best of the authors’ knowledge, this is the first prospectively registered systematic review of MEG studies focused on adult mTBI. This review has not identified sufficient evidence to support routine clinical use of MEG in mTBI currently. This is due to study heterogeneity, a lack of diagnostic test accuracy studies, and underpowered longitudinal studies of low quality. Despite this, some key areas of progress have been identified. These include the two most promising biomarkers of excess resting state low frequency power, and connectivity changes in all frequency bands. These may represent biomarkers, with potential for diagnostic application, which reflect time-sensitive changes, or may be capable of offering clinically relevant prognostic information. Verifying these findings would help meet an urgent clinical need within civilian, sports and military medicine to identify and characterise mTBI, and to allocate neurorehabilitation resources of differing nature, complexity and cost. This is best done with prospective clinical studies, using pre-defined protocols and drawing on the research guidelines highlighted in this review. Collaboration across sites would help standardise analysis methods and reporting, allowing quantitative comparison of findings across studies.

## References

[b0005] Alamian Golnoush, Hincapié Ana-Sofía, Combrisson Etienne, Thiery Thomas, Martel Véronique, Althukov Dmitrii, Jerbi Karim (2017). Alterations of Intrinsic Brain Connectivity Patterns in Depression and Bipolar Disorders: A Critical Assessment of Magnetoencephalography-Based Evidence. Front. Psychiatry..

[b0010] Antonakakis Marios, Dimitriadis Stavros I., Zervakis Michalis, Micheloyannis Sifis, Rezaie Roozbeh, Babajani-Feremi Abbas, Zouridakis George, Papanicolaou Andrew C. (2016). Altered cross-frequency coupling in resting-state MEG after mild traumatic brain injury. Int. J. Psychophysiol..

[b0015] Antonakakis M., Dimitriadis S.I., Papanicolaou A.C., Zouridakis G., Zervakisl M., Instrumentat I. (2016). Improving the Detection of mTBI Via Complexity Analysis in Resting - State Magnetoencephalography. 2016 IEEE International Conference on Imaging Systems and Techniques. IEEE International Conference on Imaging Systems and Techniques.

[b0020] Antonakakis M., Dimitriadis S.I., Zervakis M., Papanicolaou A.C., Zouridakis G. (2017). Altered rich-club and frequency-dependent subnetwork organization in mild traumatic brain injury: A MEG resting-state study. Front. Hum. Neurosci..

[b0025] Antonakakis M., Dimitriadis S.I., Zervakis M., Papanicolaou A.C., Zouridakis G. (2017). Reconfiguration of dominant coupling modes in mild traumatic brain injury mediated by delta-band activity: A resting state MEG study. Neuroscience.

[b0030] Antonakakis M., Dimitriadis S.I., Zervakis M., Papanicolaou A.C., Zouridakis G. (2020). Aberrant whole-brain transitions and dynamics of spontaneous network microstates in mild traumatic brain injury. Front. Comput. Neurosci..

[b0035] Bazarian J.J., Wong T., Harris M., Leahey N., Mookerjee S., Dombovy M. (1999). Epidemiology and predictors of post-concussive syndrome after minor head injury in an emergency population. Brain Inj..

[b0040] Belanger H.G., Curtiss Glenn, Demery Jason.A., Lebowitz Brian.K., Vanderploeg Rodney.D. (2005). Factors moderating neuropsychological outcomes following mild traumatic brain injury: A meta-analysis. J. Int. Neuropsychol. Soc..

[b0045] Boto E., Holmes N., Leggett J., Roberts G., Shah V., Meyer S.S., Muñoz L.D., Mullinger K.J., Tierney T.M., Bestmann S., Barnes G.R., Bowtell R., Brookes M.J. (2018). Moving magnetoencephalography towards real-world applications with a wearable system. Nature.

[b0050] Brookes M.J., Tewarie P.K., Hunt B.A.E., Robson S.E., Gascoyne L.E., Liddle E.B., Liddle P.F., Morris P.G. (2016). A multi-layer network approach to MEG connectivity analysis. NeuroImage.

[b0055] Carroll, L.J., Cassidy, J.D., Holm, L., Kraus, J., Coronado, V.G. 2001. Methodological issues and research recommendations for mild traumatic brain injury: The WHO Collaborating Centre Task Force on Mild Traumatic Brain Injury. J. Rehab. Med. (43 Suppl):113–25. 10.1080/16501960410023877 15083875

[b0060] da Costa Leodante, Robertson Amanda, Bethune Allison, MacDonald Matt J, Shek Pang N, Taylor Margot J, Pang Elizabeth W (2015). Delayed and disorganised brain activation detected with magnetoencephalography after mild traumatic brain injury. J. Neurol. Neurosurg. Psychiatry.

[b0065] Dimitriadis Stavros I., Zouridakis George, Rezaie Roozbeh, Babajani-Feremi Abbas, Papanicolaou Andrew C. (2015). Functional connectivity changes detected with magnetoencephalography after mild traumatic brain injury. NeuroImage Clin..

[b0070] Dimitriadis Stavros I., Antonakakis Marios, Simos Panagiotis, Fletcher Jack M., Papanicolaou Andrew C. (2017). Data-driven topological filtering based on orthogonal minimal spanning trees: Application to multigroup magnetoencephalography resting-state connectivity. Brain Connect..

[b0075] Diwakar Mithun, Harrington Deborah L., Maruta Jun, Ghajar Jamshid, El-Gabalawy Fady, Muzzatti Laura, Corbetta Maurizio, Huang Ming-Xiong, Lee Roland R. (2015). Filling in the gaps: Anticipatory control of eye movements in chronic mild traumatic brain injury. NeuroImage: Clinical..

[b0080] Dunkley B.T., Da Costa L., Bethune A., Jetly R., Pang E.W., Taylor M.J., Doesburg S.M. (2015). Low-frequency connectivity is associated with mild traumatic brain injury. NeuroImage Clin..

[b0085] Dunkley B.T., Urban K., Da Costa L., Wong S.M., Pang E.W., Taylor M.J. (2018). Default mode network oscillatory coupling is increased following concussion. Front. Neurol..

[b0090] Excellence NIfHaC. Head injury: assessment and early management. Clinical guideline [CG176]. 2017. 31869039

[b0095] Fries P. (2005). A mechanism for cognitive dynamics: Neuronal communication through neuronal coherence. Trends Cognit. Sci..

[b0100] Giza C.C., Hovda D.A. (2014). The new neurometabolic cascade of concussion. Neurosurgery.

[b0105] Gloor, P., Ball, G., Schaul, N. Brain lesions that produce delta waves in the EEG. Neurology. 1977;27(4):326–33. 10.1212/wnl.27.4.326 557774

[b0110] Gross J., Baillet S., Barnes G.R., Henson R.N., Hillebrand A., Jensen O., Jerbi K., Litvak V., Maess B., Oostenveld R., Parkkonen L., Taylor J.R., van Wassenhove V., Wibral M., Schoffelen J.-M. (2013). Good practice for conducting and reporting MEG research. Neuroimage.

[b0115] Hamm V., Héraud C., Cassel J.-C., Mathis C., Goutagny R. (2015). Precocious Alterations of brain oscillatory activity in Alzheimer’s Disease: A window of opportunity for early diagnosis and treatment. Front. Cell. Neurosci..

[b0120] Hari R., Baillet S., Barnes G., Burgess R., Forss N., Gross J., Hämäläinen M., Jensen O., Kakigi R., Mauguière F., Nakasato N., Puce A., Romani G.-L., Schnitzler A., Taulu S. (2018). IFCN-endorsed practical guidelines for clinical magnetoencephalography (MEG). Clin. Neurophysiol. Off. J. Int. Feder. Clin. Neurophysiol..

[b0125] Head J. (1993). Definition of mild traumatic brain injury. J. Head Trauma Rehab..

[b0130] Huang MX, Huang CW, Harrington DL, Nichols S, Robb-Swan A, Angeles-Quinto A, et al. Marked Increases in Resting-State MEG Gamma-Band Activity in Combat-Related Mild Traumatic Brain Injury. Cerebral cortex (New York, NY : 1991). 2019. 10.1093/cercor/bhz087 31041986

[b0135] Huang M.X., Huang C.W., Harrington D.L., Nichols S., Robb-Swan A., Angeles-Quinto A. (2019). Marked increases in resting-state MEG gamma-band activity in combat-related mild traumatic brain injury. Cereb. Cortex.

[b0140] Huang Ming-Xiong, Nichols Sharon, Robb Ashley, Angeles Annemarie, Drake Angela, Holland Martin, Asmussen Sarah, D'Andrea John, Chun Won, Levy Michael, Cui Li, Song Tao, Baker Dewleen G., Hammer Paul, McLay Robert, Theilmann Rebecca J., Coimbra Raul, Diwakar Mithun, Boyd Cynthia, Neff John, Liu Thomas T., Webb-Murphy Jennifer, Farinpour Roxanna, Cheung Catherine, Harrington Deborah L., Heister David, Lee Roland R. (2012). An automatic MEG low-frequency source imaging approach for detecting injuries in mild and moderate TBI patients with blast and non-blast causes. NeuroImage.

[b0145] Huang Ming-Xiong, Nichols Sharon, Baker Dewleen G., Robb Ashley, Angeles Annemarie, Yurgil Kate A., Drake Angela, Levy Michael, Song Tao, McLay Robert, Theilmann Rebecca J., Diwakar Mithun, Risbrough Victoria B., Ji Zhengwei, Huang Charles W., Chang Douglas G., Harrington Deborah L., Muzzatti Laura, Canive Jose M., Christopher Edgar J., Chen Yu-Han, Lee Roland R. (2014). Single-subject-based whole-brain MEG slow-wave imaging approach for detecting abnormality in patients with mild traumatic brain injury. NeuroImage Clin..

[b0150] Huang Ming-Xiong, Harrington Deborah L., Robb Swan Ashley, Angeles Quinto Annemarie, Nichols Sharon, Drake Angela, Song Tao, Diwakar Mithun, Huang Charles W., Risbrough Victoria B., Dale Anders, Bartsch Hauke, Matthews Scott, Huang Jeffrey W., Lee Roland R., Baker Dewleen G. (2017). Resting-State Magnetoencephalography Reveals Different Patterns of Aberrant Functional Connectivity in Combat-Related Mild Traumatic Brain Injury. J. Neurotrauma.

[b0155] Huang M.X., Theilmann R.J., Robb A., Angeles A., Nichols S., Drake A. (2009). Integrated imaging approach with MEG and DTI to detect mild traumatic brain injury in military and civilian patients. J. Neurotrauma.

[b0160] Huang M.X., Nichols S., Robb-Swan A., Angeles-Quinto A., Harrington D.L., Drake A. (2019). MEG working memory N-back task reveals functional deficits in combat-related mild traumatic brain injury. Cerebral Cortex..

[b0165] James S.L., Theadom A., Ellenbogen R.G., Bannick M.S., Montjoy-Venning W., Lucchesi L.R. (2019). Global, regional, and national burden of traumatic brain injury and spinal cord injury, 1990–2016: A systematic analysis for the Global Burden of Disease Study 2016. Lancet Neurol..

[b0170] Kaltiainen Hanna, Helle Liisa, Liljeström Mia, Renvall Hanna, Forss Nina (2018). Theta-band oscillations as an indicator of mild traumatic brain injury. Brain Topogr..

[b0175] Kaltiainen Hanna, Liljeström Mia, Helle Liisa, Salo Anne, Hietanen Marja, Renvall Hanna, Forss Nina (2019). Mild traumatic brain injury affects cognitive processing and modifies oscillatory brain activity during attentional tasks. J. Neurotrauma.

[b0180] Lewine J.D., Davis J.T., Sloan J.H., Kodituwakku P.W., Orrison W.W. (1999). Neuromagnetic assessment of pathophysiologic brain activity induced by minor head trauma. Am. J. Neuroradiol..

[b0185] Lewine J.D., Davis J.T., Bigler E.D., Thoma R., Hill D., Funke M. (2007). Objective documentation of traumatic brain injury subsequent to mild head trauma: Multimodal brain imaging with MEG, SPECT, and MRI. J. Head Trauma Rehab..

[b0190] Li, L.Y., Pagnotta, M.F., Arakaki, X., Tran, T., Strickland, D., Harrington, M., et al. 2015. Brain Activation Profiles in mTBI: Evidence from Combined Resting-State EEG and MEG Activity. 2015 37th Annual International Conference of the Ieee Engineering in Medicine and Biology Society. IEEE Engineering in Medicine and Biology Society Conference Proceedings. pp. 6963-6. 10.1109/EMBC.2015.7319994 26737894

[b0195] Li L, Arakaki X, Harrington M, Zouridakis G. Source Connectivity Analysis Can Assess Recovery of Acute Mild Traumatic Brain Injury Patients. Conference proceedings : . 2018;Annual International Conference of the IEEE Engineering in Medicine and Biology Society. IEEE Engineering in Medicine and Biology Society. Annual Conference. 2018:3165-8. 10.1109/EMBC.2018.8513045 30441066

[b0200] Luo Qian, Xu Duo, Roskos Tyler, Stout Jeff, Kull Lynda, Cheng Xi, Whitson Diane, Boomgarden Erich, Gfeller Jeffrey, Bucholz Richard D. (2013). Complexity analysis of resting state magnetoencephalography activity in traumatic brain injury patients. J. Neurotrauma.

[b0205] Mandal P.K., Banerjee A., Tripathi M., Sharma A. (2018;12:60-.). A comprehensive review of magnetoencephalography (MEG) studies for brain functionality in healthy Aging and Alzheimer's Disease (AD). Front. Comput. Neurosci..

[b0210] McInnes K., Friesen C.L., MacKenzie D.E., Westwood D.A., Boe S.G., Kobeissy F.H. (2017). Mild Traumatic Brain Injury (mTBI) and chronic cognitive impairment: A scoping review. PLoS One.

[b0215] Mišić Bratislav, Dunkley Benjamin T., Sedge Paul A., Da Costa Leodante, Fatima Zainab, Berman Marc G., Doesburg Sam M., McIntosh Anthony R., Grodecki Richard, Jetly Rakesh, Pang Elizabeth W., Taylor Margot J. (2016). Post-Traumatic stress constrains the dynamic repertoire of neural activity. J. Neurosci..

[b0220] Nelson L.D., Temkin N.R., Dikmen S., Barber J., Giacino J.T., Yuh E. (2019). Recovery after mild traumatic brain injury in patients presenting to US Level I trauma centers: A transforming research and clinical knowledge in traumatic brain injury (TRACK-TBI) Study. JAMA Neurol..

[b0225] Newson Jennifer J., Thiagarajan Tara C. (2019). EEG Frequency Bands in Psychiatric Disorders: A Review of Resting State Studies. Front Hum Neurosci..

[b0230] Nuwer M.R., Hovda D.A., Schrader L.M., Vespa P.M. (2005). Routine and quantitative EEG in mild traumatic brain injury. Clin. Neurophysiol..

[b0235] Oppenheimer D.R. (1968). Microscopic lesions in the brain following head injury. J. Neurol. Neurosurg. Psychiatry.

[b0240] Pang Elizabeth W., Dunkley Benjamin T., Doesburg Sam M., da Costa Leodante, Taylor Margot J. (2016). Reduced brain connectivity and mental flexibility in mild traumatic brain injury. Ann. Clin. Transl. Neurol..

[b0245] Petley Lauren, Bardouille Tim, Chiasson Darrell, Froese Patrick, Patterson Steve, Newman Aaron, Omisade Antonina, Beyea Steven (2018). Attentional dysfunction and recovery in concussion: effects on the P300m and contingent magnetic variation. Brain Inj..

[b0250] Popescu Mihai, Hughes John D., Popescu Elena-Anda, Riedy Gerard, DeGraba Thomas J. (2016). Reduced prefrontal MEG alpha-band power in mild traumatic brain injury with associated posttraumatic stress disorder symptoms. Clin. Neurophysiol..

[b0255] Popescu Mihai, Hughes John D., Popescu Elena-Anda, Mikola Judy, Merrifield Warren, DeGraba Maria, Riedy Gerard, DeGraba Thomas J. (2017). Activation of dominant hemisphere association cortex during naming as a function of cognitive performance in mild traumatic brain injury: Insights into mechanisms of lexical access. NeuroImage Clin..

[b0260] Popescu Mihai, Popescu Elena-Anda, DeGraba Thomas J., Fernandez-Fidalgo David J., Riedy Gerard, Hughes John D. (2019). Post-traumatic stress disorder is associated with altered modulation of prefrontal alpha band oscillations during working memory. Clin. Neurophysiol..

[b0265] Proudfoot M., Woolrich M.W., Nobre A.C., Turner M.R. (2014). Magnetoencephalography. Practical Neurol..

[b0270] Robb Swan Ashley, Nichols Sharon, Drake Angela, Angeles AnneMarie, Diwakar Mithun, Song Tao, Lee Roland R., Huang Ming-Xiong (2015). Magnetoencephalography slow-wave detection in patients with mild traumatic brain injury and ongoing symptoms correlated with long-term neuropsychological outcome. J. Neurotrauma.

[b0275] Rowland Jared A., Stapleton-Kotloski Jennifer R., Alberto Greg E., Rawley Justin A., Kotloski Robert J., Taber Katherine H., Godwin Dwayne W. (2017). Contrasting effects of posttraumatic stress disorder and mild traumatic brain injury on the whole-brain resting-state Network: A magnetoencephalography study. Brain Connect..

[b0280] Rowland Jared A., Stapleton-Kotloski Jennifer R., Dobbins Dorothy L., Rogers Emily, Godwin Dwayne W., Taber Katherine H. (2018). Increased small-world network topology following deployment-acquired traumatic brain injury associated with the development of post-traumatic stress disorder. Brain Connect..

[b0285] Scotland, H.I. 2020. Scottish Intercollegiate Guidelines Network checklists. Available from: https://www.sign.ac.uk/what-we-do/methodology/checklists/.

[b0290] Shah-Basak Priyanka P., Urbain Charline, Wong Simeon, da Costa Leodante, Pang Elizabeth W., Dunkley Benjamin T., Taylor Margot J. (2018). Concussion alters the functional brain processes of visual attention and working memory. J. Neurotrauma.

[b0295] Thompson, H.J., Vavilala, M.S., Rivara, F.P. 2015. Chapter 1 Common data elements and federal interagency traumatic brain injury research informatics system for TBI research. Annu. Rev. Nurs. Res.;33(1):1–11. 10.1891/0739-6686.33.1 PMC4704986 25946381

[b0300] Vakorin Vasily A., Doesburg Sam M., da Costa Leodante, Jetly Rakesh, Pang Elizabeth W., Taylor Margot J., Deco Gustavo (2016). Detecting mild traumatic brain injury using resting state magnetoencephalographic connectivity. PLoS Comput. Biol..

[b0305] van Straaten E.C.W., Stam C.J. (2013). Structure out of chaos: Functional brain network analysis with EEG, MEG, and functional MRI. Eur. Neuropsychopharmacol..

[b0310] Wilson L., Horton L., Kunzmann K., Sahakian B.J., Newcombe V.FJ., Stamatakis E.A., von Steinbuechel N., Cunitz K., Covic A., Maas A., Van Praag D., Menon D. (2021). Understanding the relationship between cognitive performance and function in daily life after traumatic brain injury. J. Neurol. Neurosurg. Psychiatry.

[b0315] Zhang Jing, Safar Kristina, Emami Zahra, Ibrahim George M., Scratch Shannon E., da Costa Leodante, Dunkley Benjamin T. (2020). Local and large-scale beta oscillatory dysfunction in males with mild traumatic brain injury. J. Neurophysiol..

[b0320] Zouridakis George, Patidar Udit, Situ Ning, Rezaie Roozbeh, Castillo Eduardo M., Levin Harvey S., Papanicolaou Andrew C. (2012). Functional connectivity changes in mild traumatic brain injury assessed using magnetoencephalography. J. Mech. Med. Biol..

[b0325] Zubarev I., Zetter R., Halme H.-L., Parkkonen L. (2019). Adaptive neural network classifier for decoding MEG signals. NeuroImage.

